# Lesion Area in the Cerebral Cortex Determines the Patterns of Axon Rewiring of Motor and Sensory Corticospinal Tracts After Stroke

**DOI:** 10.3389/fnins.2021.737034

**Published:** 2021-10-11

**Authors:** Tokiharu Sato, Yuka Nakamura, Akinori Takeda, Masaki Ueno

**Affiliations:** Department of System Pathology for Neurological Disorders, Brain Research Institute, Niigata University, Niigata, Japan

**Keywords:** brain injury, corticospinal tract, recovery, reorganization, sprouting, stroke

## Abstract

The corticospinal tract (CST) is an essential neural pathway for reorganization that recovers motor functions after brain injuries such as stroke. CST comprises multiple pathways derived from different sensorimotor areas of the cerebral cortex; however, the patterns of reorganization in such complex pathways postinjury are largely unknown. Here we comprehensively examined the rewiring patterns of the CST pathways of multiple cerebral origins in a mouse stroke model that varied in size and location in the sensorimotor cortex. We found that spared contralesional motor and sensory CST axons crossed the midline and sprouted into the denervated side of the cervical spinal cord after stroke in a large cortical area. In contrast, the contralesional CST fibers did not sprout in a small stroke, whereas the ipsilesional axons from the spared motor area grew on the denervated side. We further showed that motor and sensory CST axons did not innervate the projecting areas mutually when either one was injured. The present results reveal the basic principles that generate the patterns of CST rewiring, which depend on stroke location and CST subtype. Our data indicate the importance of targeting different neural substrates to restore function among the types of injury.

## Introduction

Brain injuries such as stroke and trauma lead to neurological deficits that frequently impair motor and sensory functions. Reports have shown that reorganization of neural networks in the spared brain region is one of the key processes to recover functions in patients, as well as in animal models (Cramer, 2008; Murphy and Corbett, 2009; Nudo, 2013; Carmichael et al., 2017; Isa et al., 2019). Brain areas and circuits that determine the recovery have been extensively studied for decades, but their contributions remain inconsistent among clinical and experimental cases. One of the factors affecting the recovery process is the location and volume of the lesion (Alexander et al., 2010; Grefkes and Ward, 2014; Isa et al., 2019; Karthikeyan et al., 2019; Aswendt et al., 2021). This suggests that the location and size of spared brain areas are the determinants of recovery, in which their circuits might be differentially reorganized in each case. However, the heterogeneity of lesion location makes it challenging to systematically understand or predict the recovery process and its neural substrate among patients. In particular, clinical studies do not usually investigate the lesion location into functional subdivisions or the relationship between neuroanatomical or physiological changes and motor outcomes (Dromerick and Reding, 1995; Edwardson et al., 2017; Hayward et al., 2017; Harvey et al., 2018). Hence, the brain areas, circuits, and their patterns of reorganization contributing to recovery are not comprehensively understood among different lesion conditions.

The corticospinal tract (CST) is one of essential neural pathways for functional recovery that connects the cerebral cortex and the spinal cord to control voluntary movements (Lemon, 2008; Boyd et al., 2017; Isa et al., 2019; Liu et al., 2021). In animal models, stroke or traumatic injury induces sprouting of spared CST axons and contributes to motor recovery (Lee et al., 2004; Ueno et al., 2012; Lindau et al., 2014; Morecraft et al., 2016; Okabe et al., 2016; Kaiser et al., 2019). In large cortical injuries of mice, CST axons originating from the contralesional cortex newly sprout to form a circuit with spinal interneurons and compensate for lost forelimb movements (Ueno et al., 2012). In similar models, increased CST sprouting by rehabilitative training, inhibition of axon growth inhibitory molecules such as Nogo, growth factors, or neural activities, have been shown to promote recovery (Lee et al., 2004; Nakagawa et al., 2013; Carmel and Martin, 2014; Wahl et al., 2014, 2017). This indicates that rewiring of the contralesional CST is essential for the recovery. Regardless of the effects in rodents, however, the importance of ipsilesional circuits, rather than contralesional circuits, is often debated (Grefkes and Ward, 2014; Cassidy and Cramer, 2017; Jones, 2017; Liu et al., 2021). In macaque monkeys, a report showed that the contralesional CST exhibited weak inputs to the motor neurons of paretic forelimbs (Zaaimi et al., 2012). The sprouting of ipsi- and contralesional projections vary depending on the cortical lesion area and size (Morecraft et al., 2016). The controversy in the involvement of two CST sources may be addressed by lesion size and location; for example, the contralesional CST may be used only when the ipsilesional CST is mostly damaged. However, the underlying neural principle that induces distinct patterns of CST rewiring in different types of cortical lesion remains unknown.

The use of variable CST pathways in the rewiring process has also not yet been examined in detail. The CST comprises multiple descending pathways originating from different cerebral cortex regions in mammals, such as rodents and primates, including humans (Coulter and Jones, 1977; Ralston and Ralston, 1985; Nudo and Masterton, 1990; Darian-Smith et al., 1996; Martin, 1996; Bareyre et al., 2002; Lemon and Griffiths, 2005; Lemon, 2008; Chenot et al., 2019). In rodents, we and others previously showed that the CST axons have different trajectories to the spinal cord from multiple cortical regions, such as the motor cortex including the rostral and caudal forelimb area [rostral forelimb area (RFA) and caudal forelimb area (CFA), respectively] and the primary somatosensory cortex (S1) (Asante and Martin, 2013; Kamiyama et al., 2015; Wang et al., 2017; Ueno et al., 2018). Each projection has distinct functions, such as the gain of reaching, grasping, and sensory processing during movement (Asante and Martin, 2013; Wang et al., 2017; Liu et al., 2018; Ueno et al., 2018). It is possible that each CST pathway compensates for each other and engages in an alternative route to command the spinal cord when another descending CST is injured. However, whether and how these multiple subtypes of projections are rewired under different injury conditions, in terms of size and location, are largely unknown. No studies have systematically examined the distinct patterns of CST rewiring among different cortical lesion types.

In this study, we developed a mouse model to induce cortical stroke in specific locations and sizes, and comprehensively investigated the rewiring patterns of the CST pathways of multiple cerebral origins. Our results provide a basic principle of rewiring patterns of CST that exhibit stroke location- and CST subtype-dependency.

## Materials and Methods

### Animals

Adult male C57BL/6J mice (Charles River) were used, which were housed under a 12-h light/dark cycle with food and water *ad libitum*. Animals were randomly assigned to a stroke or control group. All experimental procedures were performed in accordance with the protocol approved by the Institutional Animal Care and Use Committee of Niigata University.

### Surgery for Photothrombotic Stroke

**FIGURE 1:**
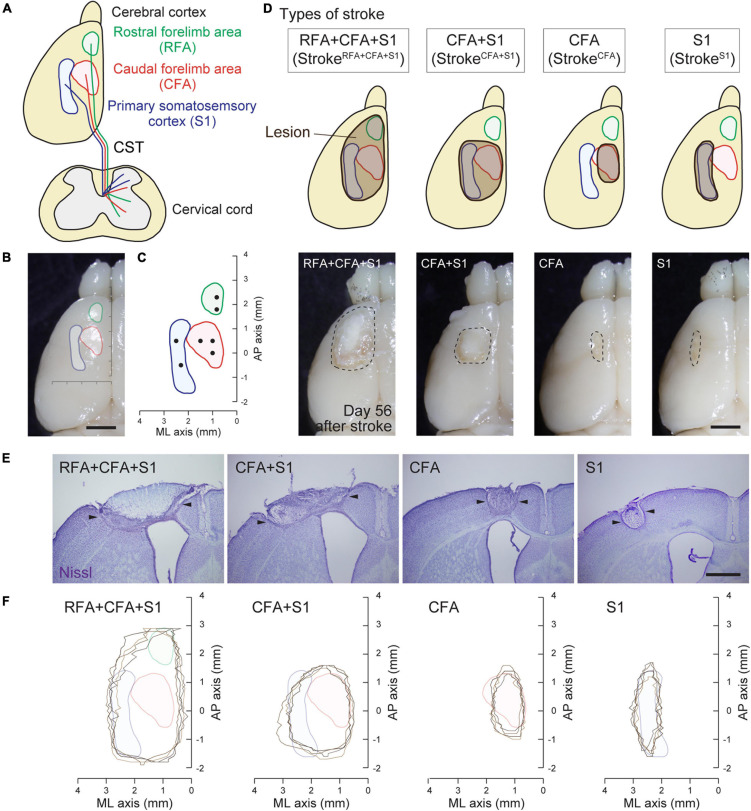
Mouse cortical stroke models in specific sensorimotor regions. **(A)** Projections of different CST pathways from the motor (RFA and CFA; green and red, respectively) and sensory cortex (S1; blue) to the cervical spinal cord. Top and transverse view of the cortex and cervical spinal cord, respectively. **(B,C)** Cortical maps showing the areas of RFA, CFA, and S1 with a dorsal view of the cerebral cortex **(B)** and AP–ML coordinates **(C)**. Black dots in **C** indicate the injection sites of anterograde tracer BDA in this study. **(D)** Four different cortical stroke models (stroke^*RFA+CFA+S1*^, stroke^*CFA+S1*^, stroke^*CFA*^, and stroke^*S1*^). Upper panels; schema of dorsal views of the cerebral cortices displaying the lesion site (brown) and the motor and sensory area. Note that the exact lesion areas are demonstrated in **F**. Lower panels; representative images of stroke lesions at day 56 after injury. The lesion areas are surrounded by dotted lines. **(E)** Representative images of the cerebral cortices of stroke models with Nissl staining in coronal sections. Arrowheads represent the lesion edges in layer V. **(F)** Horizontal maps showing the cortical lesion area of four representative animals in each stroke model. Scale bars: 2 mm in **B,D**; 1 mm in **E**.

A photothrombotic stroke was induced in the left sensorimotor cortex of 8-week-old mice as previously described (Labat-gest and Tomasi, 2013; Ueno et al., 2018). Briefly, the animals were anesthetized with isoflurane and fixed in a stereotactic frame. The skull was exposed and cleaned, and then a small piece of foil with an opening for light illumination was positioned on the left side of the skull over the targeted lesion site. To induce stroke in the (1) RFA/CFA/S1, (2) CFA/S1, (3) CFA, and (4) S1 (Figure 1D; named as stroke^*RFA+CFA+S1*^, stroke^*CFA+S1*^, stroke^*CFA*^, and stroke^*S1*^), we defined the site of opening for each stroke model as follows: (1) coordinates; mediolateral (ML) 0.5–3.5 mm and anteroposterior (AP) −2.0 to 3.5 mm from the bregma; (2) ML 0.5–3.5 mm and AP −1.0 to 2.0 mm; (3) ML 1.0–2.0 mm and AP −0.5 to 1.5 mm; and (4) ML 2.25–3.25 mm, AP −1.0 to 2.0 mm (the area was divided into two halves at AP 0.5 mm for illumination) (Table 1). Rose Bengal in saline (5 mg/ml, 50 mg/kg body weight, Sigma) was intraperitoneally injected. Five minutes after the injection, the skull was illuminated with a cold light source (90% output, CL 6000 LED, Zeiss) for the following durations: 4 min for stroke^*RFA+CFA+S1*^ and stroke^*CFA+S1*^, 5 min for stroke^*CFA*^. For stroke^*S1*^, the rostral half of the area at AP 0.5–2.0 mm was first illuminated for 4 min, then the area was covered with the foil, and the caudal half at AP −1.0 to 0.5 mm was further illuminated for 4 min. We first determined the illumination time, 5 min, for stroke^*CFA*^ model, and then found that a shorter time (4 min) was appropriate for the stroke^*RFA+CFA+S1*^ and stroke^*CFA+S1*^ models, possibly due to an increased light spread in the larger foil opening. We further determined that two illuminations of 4 min were required for stroke^*S1*^ to induce a lesion that covered the rostrocaudal axis, possibly due to an insufficient light spread compared to the larger foil openings in the stroke^*RFA+CFA+S1*^ and stroke^*CFA+S1*^ models. After light application, the scalp was sutured.

**TABLE 1:** Summary of lesion inductions in the photothrombotic stroke models.

**Type of stroke**	**Target area**	**Illumination area (coordinates)**	**Duration of illumination**	**Lesion area (average of Figure 1F)**
Stroke^*RFA*+*CFA*+*S*1^	RFA CFA S1	ML 0.5–3.5 mm AP −2.0 to 3.5 mm	4 min	ML 0.31–2.84 mm AP −1.9 to 2.9 mm
Stroke^*CFA*+*S*1^	CFA S1	ML 0.5–3.5 mm AP −1.0 to 2.0 mm	4 min	ML 0.57–2.89 mm AP −1.7 to 1.6 mm
Stroke^*CFA*^	CFA	ML 1.0–2.0 mm AP −0.5 to 1.5 mm	5 min	ML 0.73–1.85 mm AP −1.0 to 1.6 mm
Stroke^*S1*^	S1	ML 2.25–3.25 mm AP −1.0 to 2.0 mm	1st: 4 min for the rostral half (AP 0.5–2.0 mm) Second: 4 min for the caudal half (AP −1.0 to 0.5 mm)	ML 2.09–3.01 mm AP −1.6 to 1.7 mm

### Anterograde Labeling of Corticospinal Tract Fibers

Anterograde tracing was performed 42 days after the stroke or at 8-week-old for control animals (Ueno et al., 2012, 2018). Briefly, the mice were anesthetized with isoflurane and stabilized in a stereotactic frame. Then, the scalp was opened, and small injection holes were made in the corresponding sites of injection with a 27G needle. To label the CST fibers, biotinylated dextran amine [BDA; 10,000 MW; 10% weight/volume in phosphate-buffered saline (PBS); Invitrogen], an anterograde tracer, was injected into the target area of the contralesional or ipsilesional cortical hemisphere using a glass capillary attached to a Hamilton syringe. The coordinates for injection were as follows: RFA (ML 0.75, AP 1.75 mm; ML 0.75, AP 2.25 mm), CFA (ML 1.0, AP 0.0 mm; ML 1.0, AP 0.5 mm; ML 1.5, AP 0.5 mm), and S1 (ML 2.25, AP −0.5 mm; ML 2.5, AP 0.5 mm) (Figures 1C, 3A). The depth and volume of injection were 0.5 mm from the cortical surface and 0.6 μl per site, respectively. After the injection, the scalp was sutured, and the mice were returned to their home cages. The animals were perfused 2 weeks after injection (day 56 after stroke or at 10-week-old for controls) for histological analyses.

### Histology

The animals were deeply anesthetized and transcardially perfused with 4% paraformaldehyde in 0.1 M phosphate buffer. Then, the brain and cervical spinal cord were dissected out and post-fixed in the same fixative solution overnight at 4°C. The samples were cryoprotected in 30% sucrose in PBS and then embedded in an optimal cutting temperature (OCT) compound (Sakura Finetek). The brain and cervical spinal cord were cut serially into 50- and 20-μm thick coronal sections, respectively. To plot the lesion area in the sensorimotor cortex, coronal brain sections were stained with Nissl staining (0.1% cresyl violet). For BDA staining, sections of the cervical spinal cord were incubated in 0.3% Triton X-100 in PBS for 4 h, followed by Alexa Fluor 568-conjugated streptavidin (1:400, Invitrogen) in 0.1% Tween 20 in PBS for 2 h at room temperature as described previously (Ueno et al., 2012, 2018).

### Plotting of Lesion Area in the Sensorimotor Cortex

Horizontal maps representing the cortical lesion area were generated as described previously with minor modifications (Ueno et al., 2018). Briefly, images of serial cortical sections stained with Nissl were captured by an Olympus microscope (BX51) attached to a CCD camera (DP70, Olympus). Then, *X*-axis coordinates (unit = pixel) of the lesion edges in layer V and the cortical midline in each image were measured by Fiji/ImageJ software to calculate the ML axis of the lesion (Schindelin et al., 2012) (unit was changed from pixel to mm, *X* = 0 at the midline). *Y* coordinates (AP axis; unit = mm, *Y* = 0 at the bregma) were determined by the distance from the section at the bregma where the lateral ventricles begin to separate (Franklin and Paxinos, 2007). Scatterplots from the obtained XY coordinates were generated, and each plot was connected to a line to generate horizontal lesion area maps by MATLAB software (MathWorks).

### Quantification of the Number of Crossing Axons and the Density of Axons

**FIGURE 2:**
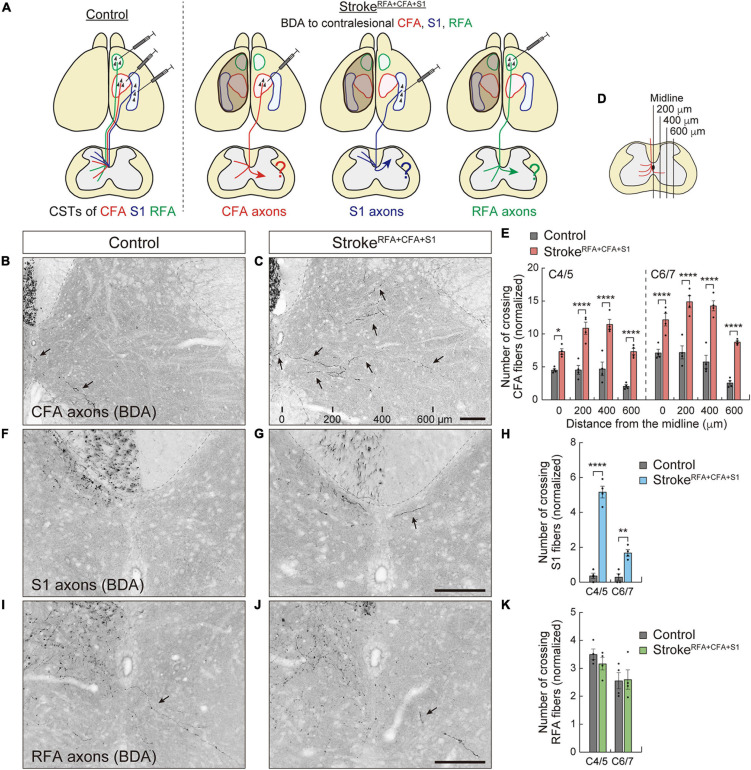
Contralesional CST axons are rewired after a large cortical stroke in RFA, CFA, and S1. **(A)** Experimental diagram of anterograde labeling of CSTs from the contralesional CFA, RFA, and S1 in stroke^*RFA+CFA+S1*^. **(B,C)** Representative images of BDA-labeled CST axons of CFA in the cervical spinal cord (C5 level) of control **(B)** and stroke^*RFA+CFA+S1*^ animals **(C)**. Arrows indicate the crossing fibers projecting to the denervated side. The distances from the midline are indicated at the bottom **(C)**. **(D,E)** The number of crossing fibers of CFA across the indicated lateral positions in the denervated side of C4–7. *F*_(1,48)_ = 290.3, *p* < 0.0001, two-way ANOVA; *p* = 0.0485 (midline at C4/5), *p* < 0.0001 (others), Bonferroni *post hoc* test. **(F,G)** Representative images of BDA-labeled CST axons of S1 in the cervical spinal cord (C5) of control **(F)** and stroke^*RFA+CFA+S1*^ animals **(G)**. **(H)** The number of crossing fibers of S1 at the midline of C4–7. *F*_(1,12)_ = 188.3, *p* < 0.0001, two-way ANOVA; *p* < 0.0001 (C4/5), *p* = 0.0019 (C6/7), Bonferroni *post hoc* test. **(I,J)** Representative images of BDA-labeled CST axons of RFA in the cervical spinal cord (C5) of control **(I)** and stroke^*RFA+CFA+S1*^ animals **(J)**. **(K)** The number of crossing fibers of RFA at the midline of C4–7. *F*_(1,12)_ = 0.2983, *p* = 0.5950, two-way ANOVA; *p* = 0.7827 (C4/5), *p* > 0.9999 (C6/7), Bonferroni *post hoc* test. *p < 0.05, **p < 0.01, ****p < 0.0001, *n* = 4. Scale bars, 100 μm.

**FIGURE 3:**
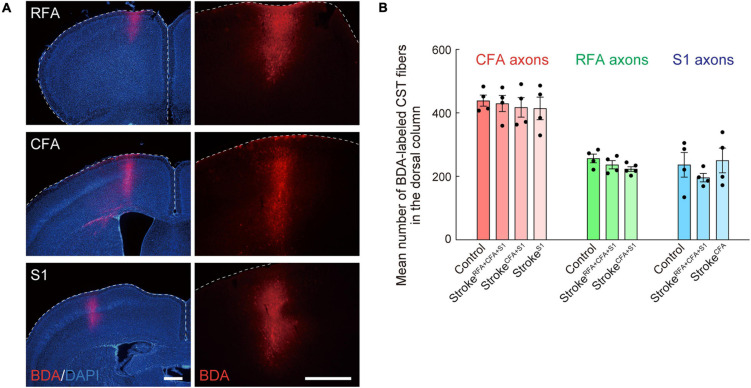
Biotinylated dextran (BDA)-labeling of CST axons of CFA, RFA, and S1. **(A)** Representative images of the BDA injection site in RFA, CFA, and S1. Right images are magnified views of the left ones. BDA, red; DAPI, blue. Scale bars, 500 μm. **(B)** Mean numbers of CST axons of CFA, RFA, and S1 in the dorsal column (C4) of control, stroke^*RFA+CFA+S1*^, stroke^*CFA+S1*^, stroke^*CFA*^, and stroke^*S1*^ animals, which were used for normalization in Figures 2E,H,K, 4D,K,L, 5E,J.

**FIGURE 4:**
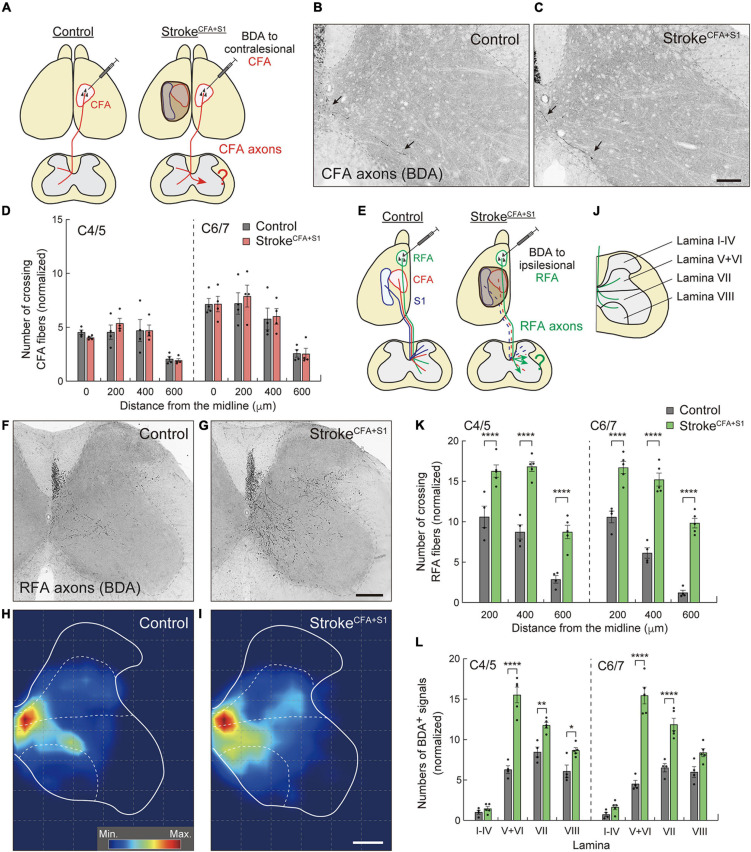
Ipsilesional CST axons of RFA are dominantly rewired after a small cortical stroke in CFA and S1. **(A)** Experimental diagram of anterograde tracing of CST axons from the contralesional CFA in stroke^*CFA+S1*^. **(B,C)** Representative images of BDA-positive axons in the cervical spinal cord (C5) of control **(B)** and stroke^*CFA+S1*^ animals **(C)**. Arrows indicate the crossing fibers projecting to the denervated side. **(D)** Quantitative data of the crossing fibers across the indicated positions in the denervated side of C4–7. *F*_(1,48)_ = 0.1689, *p* = 0.6830, two-way ANOVA; *p* > 0.9999 (all), Bonferroni *post hoc* test. **(E)** Diagram of anterograde tracing of CST axons from the ipsilesional RFA. The dotted lines indicate degenerated CST pathways from CFA and S1. **(F,G)** Representative images of CST projections of ipsilesional RFA at C5 in control **(F)** and stroke^*CFA+S1*^ animals **(G)**. **(H,I)** Distribution heatmaps of the ipsilesional CST axons of RFA in the indicated animals. **(J)** Illustration of the cervical spinal cord representing the quantified areas of axon density corresponding to laminae I–IV, V + VI, VII, and VIII. **(K)** The number of crossing fibers across the indicated positions in the denervated side of C4/5 and C6/7. *F*_(1,42)_ = 290.3, *p* < 0.0001, two-way ANOVA; *p* < 0.0001 (all), Bonferroni *post hoc* test. **(L)** The density of sprouting CST axons of ipsilesional RFA in each lamina. *F*_(1,56)_ = 290.3, *p* < 0.0001, two-way ANOVA; *p* > 0.9999 (lamina I–IV of C4/5 and C6/7), *p* < 0.0001 (lamina V–VI of C4/5 and C6/7, lamina VII of C6/7), *p* = 0.0025 (lamina VII of C4/5), *p* = 0.0327 (lamina VIII of C4/5), *p* = 0.0605 (lamina VIII of C6/7), Bonferroni *post hoc* test. *p < 0.05, **p < 0.01, ****p < 0.0001, *n* = 4 (**D**, control group in **K,L**), *n* = 5 (stroke group in **K,L**). Scale bars: 100 μm in **B,C**; 200 μm in **F–I**.

To quantify CST axon rewiring, we counted the number of crossing fibers and measured the axonal density at each cervical spinal cord section from each experimental group (10 sections at 100 μm intervals per cervical level, C4–7) in a non-blinded manner. The crossing axons of CFA in stroke^*RFA+CFA+S1*^ were further re-counted blindly. The cervical levels were confirmed by referring to the morphology of each level in the mouse spinal cord atlas (Watson et al., 2009). The data for C4 and C5 or C6 and C7 were combined for statistical analysis. To normalize BDA-labeling differences among the animals, the quantified data were divided by the mean number of BDA-labeled CST fibers in the dorsal column at C4 in two serial sections (Figure 3B). The number of crossing fibers across each vertical line at 0, 200, 400, and 600 μm from the midline was counted (Figure 2D). For measurements of the axonal density, Fiji/ImageJ software was used. First, regions of interest (ROIs) were manually defined for laminae I–IV, V–VI, VII, and VIII in each image according to the mouse spinal cord atlas (Watson et al., 2009; Figure 4J). Then, the threshold was adjusted to maintain BDA-positive signals, and the images were binarized. The number of pixels corresponding to BDA-positive signals was measured in each lamina.

### Heatmap for the Axonal Density

Regions of interest were manually defined at the cervical hemicord in each image (10 images per cervical level, C4–7). The images were then processed and binarized as described above. The XY coordinates of pixels corresponding to BDA-labeled signals were measured by using Fiji/ImageJ. The coordinates of the central canal positions were also measured to determine the reference points of the coordinates of the images. The adjusted XY coordinates of each image were combined at each cervical level and were calculated to generate a contour heatmap for axonal density by MATLAB software.

### Statistics

Statistical analyses were performed with GraphPad Prism 9 software (GraphPad Software). Statistical differences were calculated by two-way ANOVA followed by the Bonferroni *post hoc* test. All the data are represented as mean ± SEM and significant differences were considered as *p* < 0.05. All the statistical values are described in the figure legends.

## Results

### Establishment of a Mouse Cortical Stroke Model in the Specific Sensorimotor Area

We previously reported that CST axons have different trajectories to the spinal cord from the motor (RFA and CFA) and sensory area (S1) (Ueno et al., 2018; Figures 1A–C). To comprehensively examine the rewiring patterns of multiple CST pathways after the injury of the cortical area where each CST is located, we first developed a stroke model that damaged specific sensorimotor areas of the cortex in RFA/CFA/S1, CFA/S1, CFA, and S1. We selected a photothrombotic stroke model because of the controllable size and location of the lesion (Watson et al., 1985; Labat-gest and Tomasi, 2013). We first examined the size and position of the opening of the foil and illumination strength and time, and determined four different conditions to induce stroke in the RFA/CFA/S1, CFA/S1, CFA, and S1, which we named stroke^*RFA+CFA+S1*^, stroke^*CFA+S1*^, stroke^*CFA*^, and stroke^*S1*^, respectively (see section “Materials and Methods” for details; Figure 1D and Table 1). We determined that the lesion was an appropriate size and position on the brain surface at day 56 after stroke (Figure 1D, lower panels). We then created serial sections of the cerebral cortex to generate a horizontal lesion area map representing the mediolateral and rostrocaudal edges of the damaged layer V, in which the CST neurons are located (Figures 1E,F; *n* = 4 in each model). We assessed the completeness of the lesions by comparing the lesion area with the coordinates of RFA/CFA/S1 areas that we previously determined by retrograde tracing of CST neurons (Figure 1C; Ueno et al., 2018). The maps indicate that most of the target areas were appropriately damaged, and the lesion size was similar among the animals. We confirmed that stroke^*RFA+CFA+S1*^ and stroke^*CFA+S1*^ induced deficits in skilled forelimb reaching in other sets of animals (data not shown). CFA and S1 lesion are also reported to induce abnormal forelimb movements such as off target and short reaching, clumsy grasping vs. hypometria and prolonged pellet release, respectively (Ueno et al., 2018).

### Contralesional Corticospinal Tract Axons Cross the Midline and Sprout Into the Denervated Cervical Spinal Cord After a Large Cortical Lesion

The established stroke models were used to investigate the rewiring patterns of the CST pathways. A previous study demonstrated that contralesional CST axons derived from the CFA (we called this pathway motor CST) (Ueno et al., 2018) spontaneously sprout and innervate the denervated side of the cervical spinal cord after a large cortical injury, including the RFA, CFA, and S1 (Ueno et al., 2012). However, little is known about how the CST pathways from different cortical regions are rewired in the cervical level. We first induced a large stroke, stroke^*RFA+CFA+S1*^, in the left sensorimotor cortex (Figure 2A). We injected an anterograde tracer, BDA, into the contralesional CFA and analyzed the labeled CST fibers in the denervated side of the cervical spinal cord (C4–7) at day 56 poststroke (Figure 3). We observed many BDA-labeled motor CST fibers crossing the midline from the intact to the denervated side, which is consistent with previous reports (Lee et al., 2004; Ueno et al., 2012; Lindau et al., 2014; Wahl et al., 2014; Kaiser et al., 2019). The crossing fibers elongated in the lateral direction compared to the control animals (Figures 2B,C). The number of crossing fibers across the 0, 200, 400, and 600 μm positions from the midline significantly increased at both C4/5 and C6/7 levels [vs. controls; *F*_(1,48)_ = 290.3, *p* < 0.0001, two-way ANOVA; *p* = 0.0485 at 0 μm of C4/5, *p* < 0.0001 in others, Bonferroni *post hoc* test, *n* = 4; Figures 2D,E]. A blinded re-evaluation further showed similar differences [*F*_(1,48)_ = 224.3, *p* < 0.0001, two-way ANOVA; *p* = 0.0496 at 0 μm of C4/5, *p* = 0.0002 at 600 μm of C4/5, *p* = 0.0006 at 0 μm of C6/7, *p* < 0.0001 in others, Bonferroni *post hoc* test].

We next examined the rewiring of CSTs derived from the RFA or S1 (we called this sensory CST) (Ueno et al., 2018; Figures 2A, 3). Although CST fibers of S1 crossing the midline were rarely observed, the numbers significantly increased at both C4/5 and C6/7 (vs. controls, *p* < 0.0001 in C4/5, *p* = 0.0019 in C6/7, *n* = 4; Figures 2F–H). Most of the axons elongated within the 200 μm distance from the midline. In contrast, the CST fibers of RFA did not show significant changes (*n* = 4 in each group; Figures 2I–K). The data indicate that contralesional motor CST axons are extensively rewired and sensory CST axons are modestly rewired, whereas those of RFA do not significantly change.

### Contralesional Corticospinal Tract Axons Do Not Sprout Into the Denervated Cervical Spinal Cord in a Small Cortical Lesion

A large volume of lesion induced axon sprouting of the contralesional motor CST. However, whether lesion size or location affects sprouting patterns remains unclear. To reveal this, we induced a smaller stroke size, including the CFA and S1 (stroke^*CFA+S1*^) in the left cortex (Figure 4A). We again injected BDA into the contralesional CFA and analyzed the BDA-labeled fibers in the denervated cervical spinal cord. In contrast to stroke^*RFA+CFA+S1*^, we found that sprouting fibers were not clearly seen in stroke^*CFA+S1*^ mice (Figures 4B,C). The number of crossing fibers in each vertical position did not increase at either the C4/5 or C6/7 levels (Figure 4D). The data indicate that the size and location of lesions in the sensorimotor cortex are critical for inducing the sprouting of the contralesional motor CST.

### Ipsilesional Corticospinal Tract Axons Originated From the Rostral Forelimb Area Are Rewired in a Small Cortical Lesion

Previous studies reported that RFA and CFA control the same types of forelimb muscles, and their CST trajectories partially overlap (Tennant et al., 2011; Wang et al., 2017; Ueno et al., 2018). This raises the possibility that the spared CST from the ipsilesional RFA compensates for the lost CFA projections in stroke^*CFA+S1*^. Thus, we next examined whether the CST axons of the ipsilesional RFA were rewired in the denervated cervical spinal cord. We injected BDA into the ipsilesional RFA in stroke^*CFA+S1*^ and compared the projection pattern of CST axons of RFA in both control and stroke^*CFA+S1*^ mice (Figures 4E–I). In controls, BDA-labeled axons were mainly projected to the medioventral area in laminae V–VIII (Figures 4F,H,J). The CST axons of stroke^*CFA+S1*^ mice exhibited a projection pattern similar to that of control animals, in which they mostly projected in laminae V–VIII. However, the axons tended to project more laterally in laminae V–VIII, particularly near the dorsolateral column in laminae V–VI (Figures 4G,I). Moreover, axonal density appeared to be higher in laminae V–VI, VII, and VIII. We counted the number of fibers that crossed the 200, 400, and 600 μm positions and found that they significantly increased at both C4/5 and C6/7 levels after stroke^*CFA+S1*^ (vs. control, *p* < 0.0001, *n* = 4 for control, *n* = 5 for stroke^*CFA+S1*^; Figure 4K). In addition, axonal density dramatically increased in laminae V–VI of C4/5 and C6/7 (vs. controls, *p* < 0.0001; Figure 4L). The density also increased in lamina VII of C4–7 and lamina VIII of C4/5 (vs. controls, *p* = 0.0025 in C4/5 lamina VII, *p* < 0.0001 in C6/7 lamina VII, *p* = 0.0327 in C4/5 lamina VIII). In contrast, we did not detect changes in laminae I–IV, the area that sensory CST axons mainly project (Ueno et al., 2018). These results suggest that the spared ipsilesional RFA-derived CST axons sprout to compensate for the lost CFA connections in stroke^*CFA+S1*^.

### Motor and Sensory Corticospinal Tract Axons Do Not Compensate Mutually by Axon Sprouting

**FIGURE 5:**
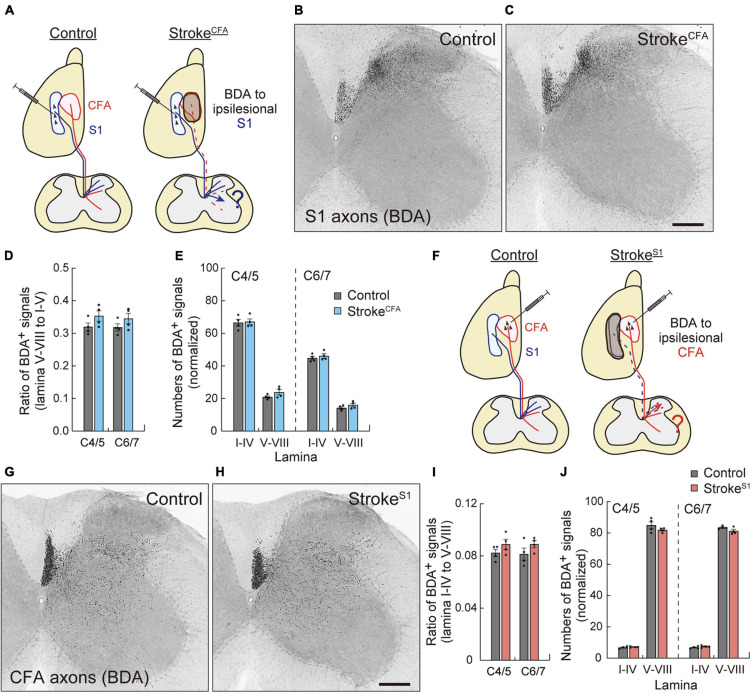
Motor and sensory CST axons are not compensatorily rewired in the stroke of S1 and CFA. **(A)** Experimental diagram of the anterograde tracing of CST from ipsilesional S1 in stroke^*CFA*^. The dotted lines indicate degenerated motor CST axons. **(B,C)** Representative images of BDA-labeled ipsilesional sensory CST axons in the cervical spinal cord (C5) of control **(B)** and stroke^*CFA*^ animals **(C)**. **(D)** The ratio of the axonal density of ipsilesional sensory CST fibers in the medioventral laminae V–VIII to the dorsal laminae I–IV. *F*_(1,12)_ = 4.691 *p* = 0.0512, two-way ANOVA; *p* = 0.2283 (C4/5), *p* = 0.3981 (C6/7), Bonferroni *post hoc* test. **(E)** The axonal density of ipsilesional sensory CST fibers in the indicated laminae at C4/5 and C6/7. *F*_(1,24)_ = 2.786, *p* = 0.1081, two-way ANOVA; *p* > 0.9999 (lamina I–IV of C4/5 and C6/7, lamina V–VIII of C6/7), *p* = 0.5789 (lamina V–VII of C4/5), Bonferroni *post hoc* test. **(F)** Experimental diagram of anterograde tracing of ipsilesional motor CST in stroke^*S1*^. **(G,H)** Representative images of BDA-labeled ipsilesional motor CST axons in the cervical spinal cord (C5) of control **(G)** and stroke^*S1*^ animals **(H)**. **(I)** The ratio of the axonal density of ipsilesional motor CST fibers in the dorsal laminae I–IV to the medioventral V–VIII. *F*_(1,12)_ = 3.860, *p* = 0.0730, two-way ANOVA; *p* = 0.4423 (C4/5), *p* = 0.3252 (C6/7), Bonferroni *post hoc* test. **(J)** The axonal density of ipsilesional motor CST fibers in the indicated laminae at C4/5 and C6/7. *F*_(1,24)_ = 2.678, *p* = 0.1148, two-way ANOVA; *p* > 0.9999 (lamina I–IV of C4/5 and C6/7), *p* = 0.1403 (lamina V–VIII of C4/5), *p* = 0.4001 (lamina V–VIII of C6/7). *N* = 4. Scale bars, 200 μm.

Next, we asked whether sensory or motor CST axons were rewired to compensate for each other. We investigated axon sprouting of the ipsilesional sensory or motor CST after stroke in the motor or sensory cortex. First, we induced stroke^*CFA*^ in the left cortex, and BDA was injected into the ipsilesional S1 to investigate the sensory CST projections (Figure 5A). In control animals, most BDA-labeled sensory CST fibers were observed in the dorsal region of the cervical spinal cord in laminae III and IV, while the axonal density was lower in laminae V–X (Figure 5B). In stroke^*CFA*^, the projection patterns appeared similar to those in the control mice (Figure 5C). We measured the axonal density in laminae I–IV, V–VI, VII, and VIII of the denervated side and did not detect significant differences in the ventral shift of density (V–VIII) from the dorsal laminae (I–IV) (*p* = 0.2283 in C4/5, *p* = 0.3981 in C6/7, *n* = 4; Figure 5D). In addition, the axonal density within laminae I–IV was not significantly changed (*p* > 0.9999 in C4/5 and C6/7, *n* = 4; Figure 5E). The data indicate that sensory CST fibers are not rewired to innervate the medioventral area where the motor CST originally projects.

Finally, we examined the sprouting of the spared motor CST after stroke in the sensory cortex. Mice were induced with stroke^*S1*^ on the left side, and the ipsilesional motor CST was labeled with BDA (Figure 5F). We observed BDA-labeled motor CST axons in laminae IV–X, with higher density in laminae V–VI and VII in both control and stroke^*S1*^ mice (Figures 5G,H; Ueno et al., 2018). Quantitative data showed that the ipsilesional motor CST fibers did not shift their projections from the ventral (V–VIII) to dorsal (I–IV) side after stroke^*S1*^ (*p* = 0.4423 in C4/5, *p* = 0.3252 in C6/7, *n* = 4; Figure 5I). The density in laminae V–VIII also did not change (*p* = 0.1403 in C4/5, *p* = 0.4001 in C6/7, *n* = 4 in each group; Figure 5J). Taken together, the results suggest that the ipsilesional motor and sensory CST have a poor potential to compensate for each other.

## Discussion

**FIGURE 6:**
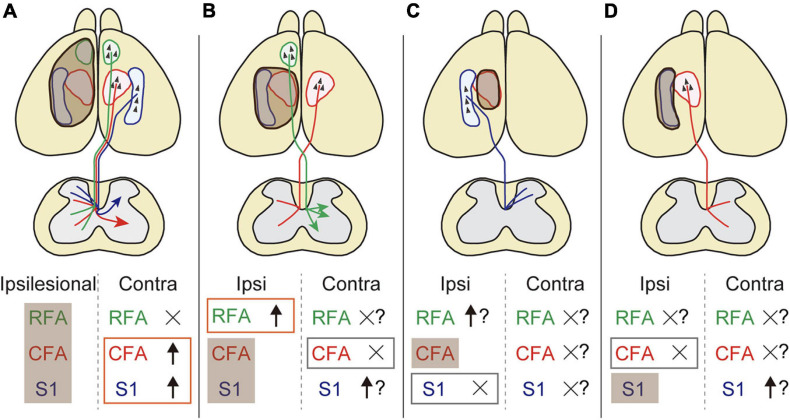
The rewiring patterns of multiple CSTs in different stroke sizes and locations. **(A)** A large stroke that damages the sensorimotor area induces axon rewiring of the contralesional motor and sensory cortex on the denervated side of the cervical spinal cord. **(B)** The remnant ipsilesional CST axons that are functionally homologous are preferentially sprouted rather than contralesional ones in a small stroke. **(C,D)** Functionally non-homologous CST pathways cannot compensate mutually. Shaded brown boxes indicate the lesion area. Orange and gray boxes are the stroke-induced sprouting and non-sprouting CST pathways that are investigated in this study, respectively. Question marks mean putative involvement in rewiring based on the presumptive rules presented in this study.

Despite the increasing knowledge about neural reorganization in recovery, we are beginning to understand the exact neural substrates across different injury conditions in terms of size and location. In this study, we systematically revealed the rewiring patterns of multiple CST pathways of different cerebral origins in various stroke conditions. We demonstrated the followings: (1) contralesional motor and sensory CST axons sprout in the denervated cervical spinal cord after a large stroke, (2) the CST axons of ipsilesional RFA arborize in the denervated side when the CFA is injured, and (3) the ipsilesional motor or sensory CST axons do not innervate the opponent territory when either one is injured (Figure 6).

We first showed that the contralesional motor CST axons grew into the denervated cervical spinal cord when RFA, CFA, and S1 were injured. This is consistent with previous studies using a similar large injury model (Lee et al., 2004; Ueno et al., 2012; Nakagawa et al., 2013; Lindau et al., 2014; Kaiser et al., 2019), though our study is the first to carefully target specific cortical regions to damage the motor and sensory CST neurons. This led to the identification of the lesion condition that increased the crossing of CST axons. Interestingly, keeping only the RFA area intact in a small stroke^*CFA+S1*^ diminished the innervation of crossing fibers, indicating that the cortical locations spared (and injured) are crucial for changing the rewiring patterns. One possibility that eliminates the crossing phenotype is that the spared ipsilesional CST pathway may compensate for the lost circuits. Indeed, the ipsilesional CST of RFA increased arborization in the region where the normal CFA-derived CST abundantly innervates (Ueno et al., 2018), suggesting that CST axons of RFA were recruited for compensation. Strokes of relatively smaller size have been shown to reorganize the perilesional area in the cortex and change cortical topography (Dancause et al., 2005; Li et al., 2010, 2015; Overman et al., 2012; Clarkson et al., 2013). In CFA lesions, several rat models demonstrate that spared ipsilesional RFA contributes to motor recovery (Nishibe et al., 2015; Okabe et al., 2016). These results suggest that the rewired CST axons of RFA might be one of the sources for the recovery.

Our data further indicate that rewiring occurs in the homologous CST population in terms of innervation area and functions (e.g., ipsi and contralesional CFA or S1, CFA, and RFA, etc.). In contrast, non-homologous circuits, especially the motor and sensory CST, appear to be difficult to compensate. They did not innervate the collaterals to the projection area of the other. Sensory and motor/pre-motor CST originally have different trajectories, connectivity, and functions. We previously showed that the motor CST projects medioventrally and connects with motor-related spinal interneurons such as Chx10^+^ V2a neurons, whereas sensory CST projects dorsally and connect with sensory-related interneurons such as the Vglut3^+^ population (Ueno et al., 2018). The motor CST functions to gain motor outputs, whereas the sensory CST engages in sensory transmission (Liu et al., 2018; Ueno et al., 2018). Furthermore, CFA and S1 have different cortical and subcortical networks (Edwards et al., 2019; Yamawaki et al., 2021). The difference in basic connectivity and neural activity may make it difficult to compensate for each pathway and function. A previous study showing that the output properties of the motor cortex cannot expand to the sensory area in motor stroke supports this notion (Harrison et al., 2013). In this case, other descending motor pathways such as the motor axons from the brain stem, and sensory afferent fibers may compensate for the denervated spinal circuits of the motor and sensory CST, respectively (Tan et al., 2012; Bachmann et al., 2014).

In this context, cortico-brainstem-spinal cord pathways may be the alternative routes for recovery. Layer V neurons have multiple collateral targets in the brain stem, such as the red nucleus and reticular formation (Kita and Kita, 2012; Muñoz-Castañeda et al., 2020). Several rodent and primate models have shown that plastic changes in the corticoreticular and corticorubral tracts are correlated or responsible for functional recovery (Bachmann et al., 2014; Ishida et al., 2016, 2019; Darling et al., 2018; Isa et al., 2019). The descending axons of the brainstem to the spinal cord may further become a source of recovery. For example, rubrospinal and reticulospinal axons innervate the cervical gray matter where the motor CST targets, and control the forelimb movements (Whishaw et al., 1998; Raineteau et al., 2001; Liang et al., 2012, 2016; Ruder et al., 2021). One study showed rewiring in reticulospinal innervation in a large stroke model (Bachmann et al., 2014). These circuits among different stroke conditions should be further explored to understand the principle that establishes compensatory pathways.

The underlying neural and molecular mechanisms may be the targets of interest in understanding the patterns of rewiring. Neurotrophic and growth factors have been explored to control motor CST rewiring, although the central mechanisms remain under investigation (Ueno et al., 2012; Jin et al., 2015; Kaiser et al., 2019). Genes and molecules that control axon sprouts, guidance, or connections may be shared in the motor/pre-motor and sensory circuits, respectively. The different projection areas and connections of the motor and sensory CSTs imply distinct molecular programs for circuit formation. A previous study showed that the motor CST could sprout into the dorsal sensory area of the spinal cord by anti-Nogo antibody treatment, which is higher than the ability of the sensory CST to innervate the ventral motor area (Bareyre et al., 2002). In another study, deletion of semaphorin signals increased ventral innervation of the sensory CST but relatively lower dorsal innervation of the motor CST (Gu et al., 2019). These results suggest that the motor and sensory CST have different molecular features as well as connectivity. It may be argued that the motor CST innervating the sensory side or vice versa could make functional connections: even if they aberrantly promote axon elongation, such as by molecular manipulation, unsynchronized neural activity in input and output neurons may make it difficult to establish functional circuits. Thus, targeting an appropriate homologous circuit is important to reconstruct functional connections for recovery.

A recent paper mentions that lesion location, volume, and CST integrity are potential biomarkers for stroke recovery (Boyd et al., 2017). Predictions of poststroke outcomes in patients are useful to develop more effective therapies in individuals such as the rehabilitation with appropriate intensity and dose. Prognostic factors in lesion size and location were systematically examined in a rat model (Jeffers et al., 2020). Our present study demonstrates potential neural substrates that could determine the recovery process among different CST and lesion types. This will provide the basis for targeting appropriate circuits in therapies using rehabilitation, neural stimulation, or molecular treatments. It should be noted that several studies using a monkey model reported more complicated patterns of rewiring, which exhibited some differences from our mouse study (Morecraft et al., 2016; Darling et al., 2018; Isa et al., 2019). The lesion in the primary motor cortex (M1) increases bouton formation of the contralesional CST of M1, as well as that of the ipsilesional supplementary motor cortex (M2) in the denervated cervical spinal cord. The contralesional M1 innervation tends to increase depending on the size of the lesion in the motor cortex (Morecraft et al., 2016). In contrast, a larger cortical lesion including the motor and somatosensory cortex (S1) decreased the innervation of the contralesional M1 CST, while it increased corticoreticular innervation (Morecraft et al., 2016; Darling et al., 2018). The principle that produces such a complicated pattern remains unknown. Although the absence of CST rewiring in a large cortical lesion may be related to the reduction of impaired forelimb use (Darling et al., 2021), the neural mechanisms should be explored further. Species differences in cortical areas and connections or experimental conditions may underlie it.

Since the present study only demonstrates the anatomical changes of the circuits, future studies need to assess their contributions to functional recovery. Although the stroke models used in the present study induce deficits in skilled forelimb reaching, the causal relation between the recovery from the initial deficits and the CST rewiring should be examined in the future. It should be noted that reorganization of the motor circuit occurs relatively in a later phase post-injury, although the scores in behavioral tests are often improved significantly in the first 1–2 weeks. For example, the number of sprouting CST fibers begins to increase at day 7–14 and peaks at day 28 (Ueno et al., 2012), and the motor map of RFA is reorganized after 1 month following the lesion (Nishibe et al., 2015). This suggests that the initial recovery may be mediated by other mechanisms, such as recovery from shock/hypometabolism/diaschisis, a tissue repair process, synaptic remodeling, disinhibition of alternative circuits, and compensatory movements. Reorganization of the motor circuits may recover fine portions of sensorimotor functions in a later phase, which should be evaluated in detailed kinematic analyses.

The present study has several caveats for interpretation. First, some of the stroke models did not damage the corresponding cortical areas completely. Although the complex shape of the areas made it difficult to precisely target them without damages in other areas due to technical limitations, further improvements in illumination area and times are needed. Since the concentration of rose bengal increases in the blood plasma up to 90 min after intraperitoneal injections (Silva et al., 2005), serial illuminations such as in the stroke^*S1*^ model may affect the lesion volume. Genetic manipulation of specific regions or cell types may overcome it. Second, we did not include a sham control group in the analyses. Because stroke^*CFA+S1*^, stroke^*CFA*^, and stroke^*S1*^ mice did not exhibit changes in sprouting compared to controls, the surgery itself might have minimal effects on the CST rewiring. However, it requires appropriate sham controls for all the rewiring patterns that were shown in the present study. Third, we did not use age-matched controls in the experiments of BDA-labeling (10-week-old for controls vs. 16-week-old for strokes). Since the number of crossing CST fibers reaches a plateau after P21 in control mice (Omoto et al., 2010), the range of ages used in this study would not much affect the numbers. Other factors such as sex differences should also be considered. Fourth, the analyses were performed in a non-blinded manner. Our blind re-evaluation showed similar differences of crossing CFA fibers in stroke^*RFA+CFA+S1*^. Although this suggests minimal bias in the analyses, we cannot exclude possible bias in the other measurements.

In summary, this study provides a conceptual scheme of the rewiring pattern of multiple CSTs after stroke in a mouse model (Figure 6). The data reveal several basic principles that generate patterns of rewiring. First, functionally homologous CSTs can compensate for each other, whereas non-homologous CSTs do not. Second, ipsilesional CSTs are predominantly used compared to contralesional CSTs. Third, contralesional CSTs are involved when ipsilesional CSTs are eliminated by the injury. The presumptive rules presented here will help to understand how the multiple pathways are rewired and intermingled to restore circuits and functions. Further investigations of different lesion location and CST subtypes, for example, CFA axons in RFA lesion and vice versa, will lead to an improved understanding of the rewiring principles. Other descending and ascending fibers as well as local circuits should be involved in the scheme. The molecular mechanisms will also help to understand the logic of rewiring. The anatomical changes in the circuits should be linked to the contribution to functional improvements. This will lead to identify and target appropriate neural substrates to develop clinical therapies for restoring the functions.

## Data Availability Statement

The original contributions presented in the study are included in the article, further inquiries can be directed to the corresponding author.

## Ethics Statement

The animal study was reviewed and approved by the Institutional Animal Care and Use Committee of Niigata University.

## Author Contributions

MU conceived the project. TS, YN, and MU designed and performed the experiments. TS and MU analyzed the data and wrote the manuscript. AT analyzed the axon density. All authors contributed to the article and approved the submitted version.

## Conflict of Interest

The authors declare that the research was conducted in the absence of any commercial or financial relationships that could be construed as a potential conflict of interest.

## Publisher’s Note

All claims expressed in this article are solely those of the authors and do not necessarily represent those of their affiliated organizations, or those of the publisher, the editors and the reviewers. Any product that may be evaluated in this article, or claim that may be made by its manufacturer, is not guaranteed or endorsed by the publisher.

## Abbreviations

BDA: biotinylated dextran amine
CFA: caudal forelimb area
CST: corticospinal tract
M1: primary motor cortex
RFA: rostral forelimb area
S1: primary somatosensory cortex.
